# Effects of a mobility monitoring system on the cost of care in relation to reimbursement at Swiss nursing homes: learnings from a randomized controlled trial

**DOI:** 10.1186/s13561-017-0178-y

**Published:** 2017-12-01

**Authors:** Mario Stark, Rigo Tietz, Heidrun Gattinger, Virpi Hantikainen, Stefan Ott

**Affiliations:** 1 Institute of Business Management, University of Applied Sciences St. Gallen, Rosenbergstrasse 59, Postfach, 9001 St. Gallen, Switzerland; 2 Institute for Applied Nursing Science, University of Applied Science St. Gallen, St. Gallen, Switzerland; 3 Statistician, University of Applied Sciences St. Gallen, St. Gallen, Switzerland

**Keywords:** Health economics, Cost of care, Reimbursement, Monitoring system, Nursing home

## Abstract

**Background/objective:**

Nursing homes in Switzerland are under pressure to efficiently coordinate staff activities to cover their personnel costs under the care financing system. In this study, the use of a mobility monitoring system accompanied with case conferences was investigated in order to improve sleep quality and estimate the cost benefit of this intervention.

**Method:**

In an open two-phase randomized controlled trial at three nursing homes, residents with cognitive impairment were randomly assigned to an intervention group and a control group. In the intervention group, a 10-week period of intensive use of the monitoring system and case conferences led by an advanced nurse practitioner (Phase I) was followed by 3 months of reduced use of the monitoring system and case conferences led by an internal registered nurse (Phase II). In the control group, the monitoring system was only used for data acquisition. Nurses reported the activities with a specifically developed tool. Based on the recorded activities, the cost of care was calculated. The correlating reimbursement per patient was calculated from the care levels in the Swiss reimbursement system. Data from 44 residents was included in the analysis with a linear mixed model.

**Results:**

Although analysis revealed no statistically significant effects, results indicate that the use of a monitoring system can guide nurses in organizing their tasks to increase effectiveness. Information systems such as the mobility monitor can help to identify single outliers that do not correspond with the overall situation.

**Conclusion:**

In the health care system, problematic individual cases can account for a disproportionally high cost levels. It was shown that information systems can have a significant economic impact in the long run.

**Trial registration:**

The study is registered at the German Clinical Trials Register under the Nr. DRKS-ID: DRKS00006829.

## Background

### Challenges of cost efficiency in nursing homes

**Fig. 1:**
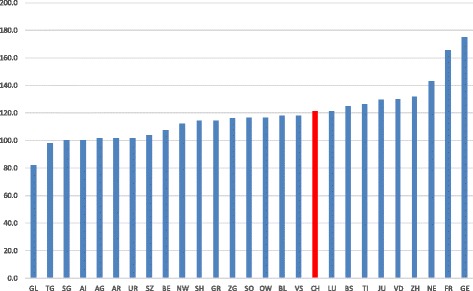
Cost of Care per Day in Switzerland. Average cost of care in nursing homes per day (y-axis) compared between the different cantons of Switzerland (x-axis). All numbers in CHF. Swiss average highlighted in red

Swiss nursing homes struggle with the cost limitation policies imposed by local and regional constituencies [1]. Large differences also exist in the costs of care per day between the different cantons (Fig. 1; source: [2]). Hence, there is room for improvement in specific institutions. Crivelli et al. [3] investigated the relationship between cost efficiency, the alternative institutional forms, and the different regulatory settings. Their results “showed that only approximately 60% of the nursing homes included in our sample operate close to the national standard for efficiency.” In an earlier study, Fillipini [4] found that economies of scale exist for most output levels at Swiss nursing homes in the Ticino. The study could not identify if the effects are more due to potential savings in the cost of direct care or in infrastructure cost.

A growing number of new technologies are becoming available within nursing care. These can improve the quality of care, reduce costs, or enhance working conditions [5]. To date, most studies have focused on the effect of electronic medical record (EMR) systems on cost and quality of care. For instance, Hitt and Tambe [6] investigated the relationship between the use of electronic medical record (EMR) systems and nursing home performance. They found a positive effect of EMR-system implementation, about 1% higher productivity, 3% greater efficiency, but approximately 2.7% higher cost. Therefore, the economic effect of the introduction of EMR might be questionable.

### New technologies for monitoring of residents

However, there are many different approaches to using new technology to improve efficiency in nursing homes.

In a study performed at a retirement community in Missouri, Rantz et al. [7] used environmentally embedded sensor systems to create automated health alerts. Results indicated that residents living with sensors were able to reside 1.7 years longer than residents living without sensors, suggesting that proactive use of health alerts facilitates successful aging. Cost estimates revealed potential savings in labor cost of about USD 30′000 per person, based on the improved coordination of care by the nurses.

Beaty and Sauer [8] showed that the use of a smart bed technology reduced new pressure ulcers by 85% in new pressure ulcer development. An additional economic analysis resulted in estimated net savings of USD 9.06 to 14.70 per bed per day.

Slight et al. [9] equipped a community hospital with a monitoring unit that measures mechanical vibrations of the heart cardio ballistic motion effect, respiratory and patient motion. “Average net benefit of implementing the system ranged from $224 per patient […] to $710 per patient.” It was therefore concluded that the implementation of this monitoring system resulted in a positive return on investment.

In the study discussed here, the use of a mobility monitoring system accompanied with case conferences was investigated to improve sleep quality in nursing home residents with cognitive impairment. The design and the sample of the study are reported in detail elsewhere [10]. While sleep quality in the intervention group increased, sleep quality in the control group remained almost stable. Results indicate that using a mobility monitoring system can improve the assessment of nighttime mobility and activity and support nurses in planning and implementing care interventions (repositioning, continence care, and inspection rounds). In parallel, the influence on cost of care was assessed. The results of these health economic evaluations are presented here.

## Methods

The study protocol was approved by the responsible ethics review committee (EKSG 14/101).

### Design

**Fig. 2:**
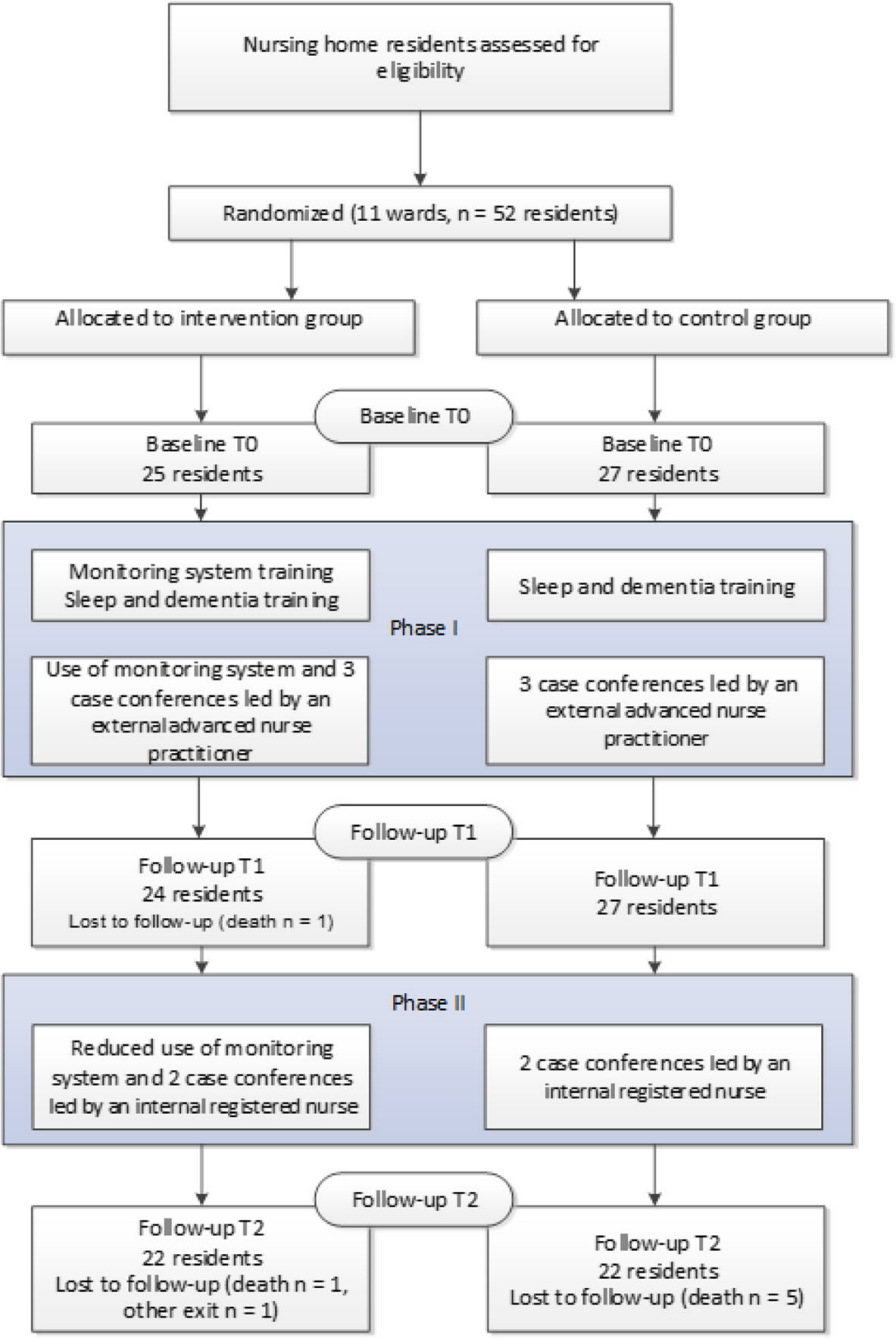
Study Flowchart. This flowchart shows the flow of interventions and data collection over the presented study as well as number of residents per study phase

This study was designed as an open two-phase randomized controlled trial. It was conducted at 11 wards in a convenience sample of three nursing homes between November 2014 and September 2015. A 10-week period of intensive use of the monitoring system and case conferences led by an advanced nurse practitioner (Phase I) was followed by 3 months of reduced use of the monitoring system and case conferences led by an internal registered nurse (Phase II). Randomization at the level of nursing home wards was chosen to prevent contamination between intervention and control participants. Simple randomization was used to assign the wards to the intervention and control group. Data collection took place at baseline (T_0_), after Phase I (T_1_ = 10 weeks after intervention started), and after Phase II (T_2_ = 6 months after intervention started). The patient flow can be seen in Fig. 2.

### Setting and participants

Three medium-sized nursing homes in German-speaking Switzerland participated in the study. Within the nursing homes, all wards and all affiliated qualified nurses and student nurses were involved. To be eligible for the study, nursing home residents had to suffer from cognitive impairment and sleeping disorders. Thirty-two women and 12 men with a mean age of 87.5 years (SD: 7.1) were included. Residents’ mean duration of stay at the beginning of the study was 40.8 months (SD: 32.9). On average, 20.5% of participants fell into care levels 1–4, 59.0% into care levels 5–8, and 20.5% into care levels 9–12. Inclusion criteria and demographic data are described in more detail in Gattinger et al. [10].

### Intervention

Interventions included in-house training on the monitoring system and interpretation of the data gathered by the system. In each institution, one or two nurses were trained as key-users to support other nurses if questions arose about the monitoring system. In addition, in-house training on dementia and sleep was provided.

The situation of each participating resident was discussed during case conferences. A case conference is defined as a goal-oriented, systematic method that team members can use to exchange professional opinions on a particular care problem. Case conferences can be a valid tool for nursing home staff to deal professionally with individual residents’ sleep disturbances. Case conferences are particularly useful for understanding difficult situations.

In the intervention group, monitoring data was included in the discussion. In Phase I, three case conferences per nursing home resident were conducted. Case conferences were led by an external advanced nurse practitioner. In phase II, two case conferences per nursing home resident led by an internal registered nurse were conducted. The discussed problems, favored outcomes, and planned interventions were recorded.

### Use of the monitoring system

The monitoring system used in this study is an in-bed system that records mobility and micro-activity without any body contact. It includes warnings in cases of immobility with individually adjustable tolerance (2 h, 3 h, 4 h). In addition, a bed-exit alarm can be activated. Data is continuously wirelessly transmitted, stored and analyzed with dedicated software.

Individual mobility profiles were used to assess resident activity and movement patterns and to support nurses’ decision whether to initiate a sleep-promoting intervention. The data and the warning system were also used to establish whether residents needed to be moved due to decubitus prophylaxis.

### Instruments used to assess care activities

Nurses’ daytime and nighttime activities were recorded using a self-developed assessment tool designed for economic assessment.

The following daytime activities were recorded:

- Changing position in bed as decubitus prophylaxis
- Transfer from or into the bed, wheelchair, or into a sitting position
- Accompanied walking in the resident’s room
- Accompanied walking outside the resident’s room
- Support with going to the toilet
- In addition, any aids used were recorded (positioning aids, security bars around the bed or bell mats)

The following nighttime activities were recorded:

- Check-up visits
- Changing position in bed as decubitus prophylaxis
- Support with going to the toilet
- Incontinence care
- Other activities as stated
- In addition, any aids used were recorded (positioning aids, security bars around the bed or bell mats)

### Cost calculation of respective care activities

To calculate the time needed for each of these activities, the average time quoted on the LEP was used [11]. The LEP is a performance classification instrument for the uniform documentation of services in the healthcare sector. This method is consistent with the International Classification of Nursing Practice (ICNP) and the International Standard Classification of Occupations (ISCO).

This information was used to calculate the time spent by nurses caring for the study participants. Finally, based on the actual personnel costs (including nursing home overheads), the total cost of the care activities was calculated (see below “cost of care”). It must be noted that in these study nurses’ activities relevant for sleep and activities connected with mobility impairment (e.g. decubitus and fall prophylaxis) were recorded. Other care activities such as support with personal hygiene or nutrition were not included and thus not reflected in the calculation.

### Calculation of reimbursement

The care needs of each participant were assessed at each data collection point (T0, T1, T2). This was done using the assessment instrument normally used at the respective nursing home. This was either BESA (BewohnerInnen EinStufungs- und Abrechnungssystem; for supplier details, see besacare.ch) or RAI-NH (Resident Assessment Instrument – Nursing Home; see rai.ch).

In the Swiss reimbursement system for elderly care in nursing homes, care levels (“Pflegestufen”) are assigned based on a scale of 1 to 12, defined by the activities needed for the specific. These care levels are based on BESA or RAI assessments and define nursing home reimbursement. For instance, the monthly reimbursement for care level 3 is currently CHF 30.10 per day.

### Data analysis

For data analysis IBM SPSS Statistics Version 23 was used. Nurses’ activities were assessed over five nights/days in T_0_, T_1_ and T_2_. For the sake of stability, the average of five measured values was used for each activity in each point of time. Sums of activities per night/day were calculated. To account for variation over time (repeated measurement component) as well as between groups (independent sample component: intervention group vs. control group) linear mixed models were employed. F-tests were used to test for differences between the groups (intervention group vs. control group), over time and for interaction. Sphericity was tested by Mauchly’s test, using the Greenhouse-Geisser modification in case of violation of Sphericity. We used Kolmogorov-Smirnov tests to assess normality of the dependent variables and calculated Cooks’s distances to find out if there are outliers. Single activities as well as the sum of activities were tested for group specific differences (intervention group vs. control group), for changes over time (T_0_, T_1_, T_2_) and for interactions (group specific evolvement over time). To assess relationships between metric or pseudometric data Pearson correlations were calculated. Tests were performed at a level of significance of 5%. For sample size calculations we used GLIMMPSE (Kreidler et al. [12]).

## Results

### Care activities

Table 1 shows nurses’ caregiving activities. Statistically, average nighttime care activities were reduced significantly in both groups during the first phase. While in the control group the number of activities between T1 and T2 remained approximately the same, the number of nighttime activities in the intervention group increased in the second phase to a level above the initial T0. The number of control visits at night exhibits a statistically significant difference between the measurement points. At T1, both groups show a marked decline in average check-up visits, whereas at T2 the number of visits increases again. Regarding incontinence care at night, a significantly lower frequency over time (T0 to T2) is shown in the control group. In the first phase, a reduction occurred in the intervention group, whereas in the second phase incontinence care again increased above the starting level at T0.

**Table 1:** Recorded activities

		F	df	p	Group	T0: MW (SEM)	T1: MW (SEM)	T2: MW (SEM)
Sum of activities	Group	0,416	1	0,523	KG	7091 (0,353)	6255 (0,411)	6245 (0,595)
	Time	5575	1670	*0,009	IG	6448 (0,361)	5048 (0,421)	7114 (0,609)
	Time*Group	4159	1670	*0,026				
Check-up visits at night	Group	1155	1	0,289	KG	3655 (0,155)	3055 (0,177)	3218 (0,178)
	Time	9297	2	*0.000	IG	3162 (0,158)	2800 (0,181)	3305 (0,182)
	Time*Group	3242	2	*0,044				
Decubitus prophylaxis at night	Group	0,044	1	0,834	KG	1309 (0,237)	1227 (0,234)	1345 (0,368)
	Time	2550	15,900	0,097	IG	1248 (0,280)	0,771 (0,240)	1648 (0,377)
	Time*Group	1475	1590	0,236				
Support with toilet at night	Group	0,066	1	0,799	KG	0.900 (0,332)	1082 (0,415)	0,836 (0,381)
	Time	0,126	2	0.882	IG	1210 (0,340)	0,848 (0,425)	1143 (0,390)
	Time*Group	1431	2	0,245				
Incontinence care at night	Group	0,895	1	0,350	KG	1227 (0,170)	0,891 (0,127)	0,845 (0,134)
	Time	3643	2	*0,031	IG	0,829 (0,174)	0,629 (0,130)	1019 (0,137)
	Time*Group	4405	2	*0,015				
Sum of activities during the day	Group	0,646	1	0,426	KG	11,127 (1385)	11,827 (1289)	12,818 (0,935)
	Time	1405	2	0,251	IG	12,829 (1319)	12,829 (1319)	13,781 (0,957)
	Time*Group	0,048	2	0,953				
Decubitus prophylaxis during the day	Group	0,568	1	0,455	KG	1882 (0,407)	1318 (0,319)	1755 (0,426)
	Time	6942	2	*0,002	IG	1990 (0,417)	1629 (0,326)	2495 (0,436)
	Time*Group	1609	2	0,206				
Transfer during the day	Group	0,520	1	0,475	KG	2609 (0,373)	2655 (0,517)	2964 (0,285)
	Time	2137	2	0,125	IG	2495 (0,382)	3724 (0,530)	2981 (0,292)
	Time*Group	2142	2	0,124				
Accompany in room during the day	Group	0,051	1	0,823	KG	2392 (0,344)	2591 (0,222)	2782 (0,251)
	Time	1046	1867	0,352	IG	2486 (0,352)	2343 (0,227)	2752 (0,257)
	Time*Group	0,238	1867	0,774				
Accompany outside room during the day	Group	0,315	1	0,578	KG	2618 (0,368)	3018 (0,373)	3118 (0,273)
	Time	0,259	2	0,773	IG	3276 (0,377)	2876 (0,381)	3162 (0,279)
	Time*Group	0,915	2	0,405				
Support toilet during the day	Group	0,626	1	0,433	KG	1627 (0,290)	2245 (0,320)	2200 (0,292)
	Time	1984	2	0,144	IG	2333 (0,297)	2257 (0,328)	2390 (0,299)
	Time*Group	2216	2	0,116				

F = F-value, df = degrees of freedom, *p* = *p*-value; MW = average, SEM = standard deviation, *p-value < 0,5

Daycare activities reveal no statistically significant changes. Only in the number of decubitus prophylaxis can a statistically significant effect be observed over time in both groups. First, there is a reduction in decubitus prophylaxis at T1, but an increase is observed at T2, which exceeds the initial value in the intervention group.

### Cost of care

Table 2 shows the average cost of care, calculated based on the activities recorded. No relevant changes occurred from T0 to T1 nor did any differences between the control group and the intervention group.

**Table 2:** Cost of Care in CHF

Average per resident per day	Activities in minutes at T0	Cost of Care in CHF at T0	Activities in minutes at T1	Cost of Care in CHF at T1
Control Group	191	124.59	200	130.47
Intervention Group	206	134.45	202	131.89

### Reimbursement

Table 3 reports average reimbursement per resident, calculated based on the care levels assessed using BESA or RAI and the Swiss reimbursement system. No significant difference was found between the two groups. However, a pronounced difference in care levels was identified between T0 and T1, driven by growing care needs over time.

**Table 3:** Reimbursement in CHF

Average per resident per day	Reimbursement at T0	Reimbursement T1
Control Group	77.95	84.61
Intervention Group	81.27	85.00

### Reimbursement and cost of care

The data shows that the level of care increased both in the intervention group and in the control group during the study. This increase is statistically significant (F = 6.040, *p* = 0.005). This means that nursing home revenues have increased. The care activities at night have also increased (F = 5.575, *p* = 0.09). Nevertheless, analysis of the recorded activities could not prove that applying the mobility monitor leads to a higher level of efficiency in the care process, which would enable relevant savings in terms of economic outcomes.

There were no significant differences in care expenses between T0 and T1 in the intervention group, as well as in the control group during the same period. Differences between the nursing homes regarding the discussed outcomes (cost of care and care levels) could be observed, but were not statistically significant due to the small sample size.

**Fig. 3:**
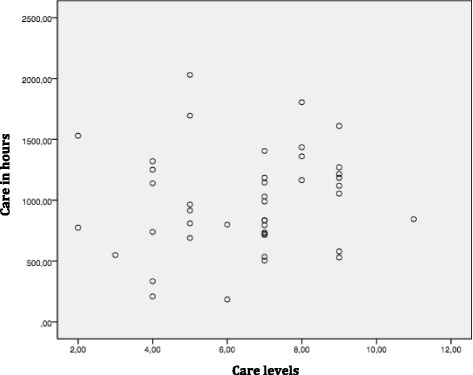
Relationship between cost of care and reimbursement, for all residents. Relationship between reimbursement, represented by the care levels (x- axis) and care in hours during 5 days/nights (y-axis), per resident

Figure 3 shows the link between care expenses/activities and care level for both the intervention group and the control group at time T0. No correlation could be found between that care level that drives reimbursement and care activities, which, in turn, drive care costs. The same holds true for T1 and T2 respectively.

### Changes in reimbursement and cost of care

**Fig. 4:**
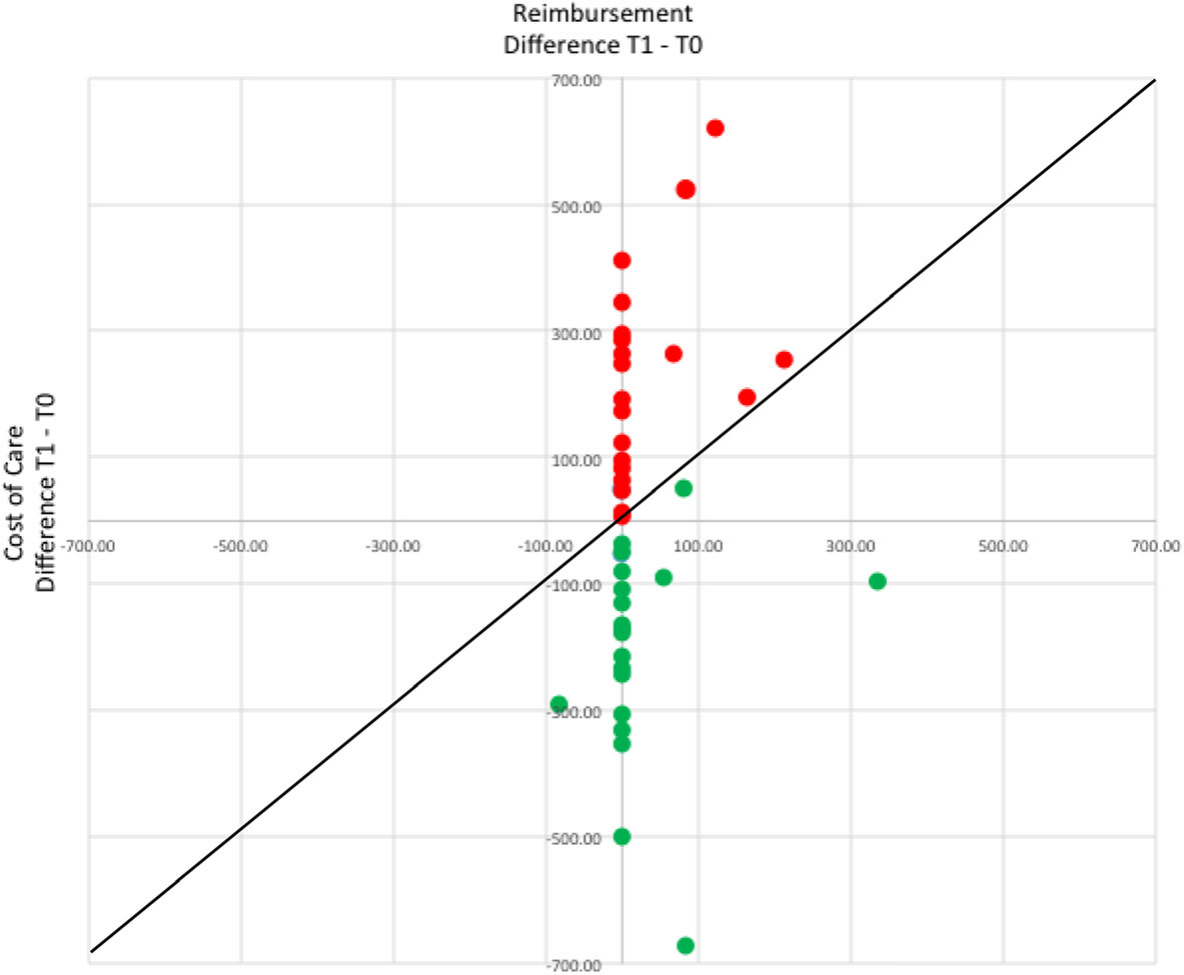
Changes in Reimbursement and Cost of Care – T0 vs. T1. Changes in cost of care from T0 to T1 versus the changes in reimbursement from T0 to T1, both in CHF. Diagonal line represents the position where additional costs were compensated with additional reimbursement

Next, the changes between care levels and care expenses between T0 and T1 were plotted. Figure 4 compares the difference in reimbursement based on the change of care levels between T0 and T1 (x-axis) and the cost of care (y-axis), calculated by multiplying the recorded hours with personnel costs. The diagonal line indicates where additional costs are compensated by additional reimbursement. Any data point above the line indicates a resident for whom costs have increased disproportionally compared to reimbursement. Any data point beneath the line indicates a resident for whom the increase in reimbursement was greater that the increase in cost. If revenue (reimbursement minus cost of care) was positive, the data point is marked in green; if negative, in red). Evident are those cases in which the economic situation per resident has improved while in others the situation has deteriorated, i.e., the respective nursing home has increased costs while revenues have remained stable.

Example:

Table 4 shows that the ratio between cost of care and reimbursement could be improved significantly in specific cases. This was not true in all cases, for instance, when the cost of care was covered by reimbursement whereas the loss per resident was reduced. For example, resident 2A10 needed care at T0 that cost CHF 149.08 per day. Given that only CHF 45.20 were reimbursed, the nursing home lost CHF 103.88 per day. At T1, the reimbursed CHF 45.20 did not cover the present cost of CHF 78.46 per day, but the loss for the nursing home was reduced by CHF 70.62 per day.

**Table 4:** Reimbursement and Cost of Care in CHF per day

	T0					T1					Difference T1-T0
Resident	Care level	Reimbursement	Care in Minutes	Cost of Care	Profit/Loss	Care Level	Reimbursement	Care in Minutes	Cost of Care	Profit/Loss	Profit/loss
2B12	5	54.70	339	221.66	−166.96	6	71.40	499	326.28	−254.88	−87.92
4C20	7	87.70	167	109.19	−21.49	7	87.70	273	178.50	−90.80	−69.31
2A08	7	87.70	281	183.73	−96.03	7	87.70	362	236.70	−149.00	−52.96
4C16	9	112.10	116	75.85	36.25	9	112.10	192	125.54	−13.44	−49.69
2A12	5	54.70	162	105.93	−51.23	5	54.70	221	144.50	−89.80	−38.58
2A05	9	112.10	224	146.46	−34.36	9	112.10	253	165.43	−53.33	−18.96
2A09	9	112.10	254	166.08	−53.98	9	112.10	274	179.16	−67.06	−13.08
1B09	8	101.20	233	152.35	−51.15	8	101.20	248	162.16	−60.96	−9.81
4C22	5	54.70	183	119.66	−64.96	5	54.70	187	122.27	−67.57	−2.62
4C17	4	45.20	42	27.46	17.74	4	45.20	27	17.65	27.55	9.81
1C02	7	87.70	159	103.96	−16.26	7	87.70	143	93.50	−5.80	10.46
4C24	7	87.70	107	69.96	17.74	7	87.70	83	54.27	33.43	15.69
4C21	5	54.70	138	90.23	−35.53	5	54.70	98	64.08	−9.38	26.15
2B11	8	101.20	287	187.66	−86.46	9	112.10	260	170.00	−57.90	28.55
4C23	9	112.10	106	69.31	42.79	9	112.10	52	34.00	78.10	35.31
1B08	7	87.70	229	149.73	−62.03	6	71.40	140	91.54	−20.14	41.89
1C01	7	87.70	198	129.46	−41.76	7	87.70	132	86.31	1.39	43.15
1B06	7	87.70	237	154.96	−67.26	7	87.70	166	108.54	−20.84	46.42
2A06	9	112.10	322	210.54	−98.44	9	112.10	229	149.73	−37.63	60.81
2A10	4	45.20	228	149.08	−103.88	4	45.20	120	78.46	−33.26	70.62
2A04	2	20.70	306	200.08	−179.38	7	87.70	277	181.12	−93.42	85.96

## Discussion

In this study, it was sought to assess the influence of the use of a monitoring system accompanied with case conferences on cost of care. The discussion on the economic impacts of applying the mobility monitor in the care process refers mainly to the cost-income balance of the examined nursing homes. The cost situation is reflected in the study by the variable care expenses. It focuses on labor costs and is operationalized by the time needed to execute care activities. Further cost factors, such as material costs and incidental expenses, are not taken into consideration because they only account for a small proportion of the overall costs and were not expected to change significantly in the study. The income situation is reflected by the level of care because this factor determines the reimbursement that nursing homes can claim from health insurances.

The analyzed data exhibit no statistically significant relationship between care expenses and care level. This finding needs to be discussed in more detail. Care expenses were measured retrospectively by the operating nurses and reflect the number of activities to be carried out. Although this measure does not fully cover all the activities of the care process, the outcomes are highly relevant and call for discussion. This is necessary because the outlined limitations apply to all patients and because this study focuses on the overall relationship between care expenses and care level rather than on single activities.

The care level reflects the ex-ante estimated amount of activities needed in the care process of an individual patient. As outlined, care-level determination follows a standardized procedure according to the BESA or the RAI scheme.

From an economic perspective, the missing link between actual care expenses and estimated needs for care activities leaves room for improvement. In current practice, the level of care for an individual patient is adjusted on a regular basis (normally every 6 months) and takes into consideration nurses’ observations and written documentation. In this context, the results raise several questions potentially relevant for current practice:

- How can the outlined evaluation process be improved? What can be done that ex ante estimations meet ex post observations? How can this fit be measured in practice?
- What would be the economic impact of an improvement? For whom?
- Missing link: Is there a systematic overestimation or underestimation? Or do discrepancies occur randomly?
- Are there incentives to reduce the care level? Are there incentives to raise it?
- Which incentives exist for nursing homes to improve the process? Is there an economic benefit of doing better?
- Might other stakeholders be affected? (e.g., insurances)

To discuss the outlined questions, case-based examples should be illustrated and discussed in more detail. Data includes several individual cases that exhibit an economically unfavorable path regarding the relation between care expenses and care level as a proxy for the income situation.

In this context, further practically and academically relevant questions can be formulated: How did the procedures and practices regarding the application of the mobility monitor differ in the examined nursing homes? What are the best practices for ensuring the most favorable outcomes?

Notably, significant interaction was observed between time and group: a more pronounced increase in sleep quality was found in the intervention group in T1 (see [10]). Therefore, not only directly cost-driven factors must be considered, but also the quality of care and residents’ subsequent quality of life.

The limitations of the study include the small study size that did not allow investigating some effects of the intervention, as well as the cost of medication that was not taken into account. Furthermore, there are limitations in the current care level evaluations systems (BESA/RAI) insofar as the care activities for residents with dementia are not well reflected in the current systems [13]. As a further limitation, the cost of additional tools like security bars or positioning aids were not taken into consideration. The cost of the medication given to residents was not assessed either, given that in the Swiss reimbursement system medication is covered by the health insurance of the individual resident.

## Conclusions

The study results and feedback from the participating nurses suggest a positive overall impact of applying the mobility monitor in the care process (e.g., improved sleep quality). These effects cannot be attributed solely to applying the mobility monitor as a supporting, technology-based information system with hardware and software components. All interventions were combined with nursing care conferences that provided inputs on the care process and on the usage of the mobility monitor device by external and internal nursing experts.

On average, these results demonstrate the presence of a high sleep quality in the examined patients and a high-level care process at the nursing homes under study. In this context, further improving the aggregated level of these measures might be very difficult and questionable in terms of economic benefits. However, information systems such as the mobility monitor can help to identify single outliers that do not correspond with the overall situation. In the health care system, problematic individual cases can account for a disproportionally high cost levels. It was shown that information systems can have a significant economic impact in the long run.
